# Psammomatoid and trabecular variants of juvenile ossifying fibroma—two case reports

**DOI:** 10.4103/0971-3026.50832

**Published:** 2009-05

**Authors:** Simi Thankappan, Sherin Nair, Valsa Thomas, KP Sharafudeen

**Affiliations:** Department of Oral Medicine and Radiology, Government Dental College, Calicut-673 008, Kerala, India; 1 Department of Oral Pathology and Microbiology, Government Dental College, Calicut-673 008, Kerala, India

**Keywords:** Cemento-ossifying fibroma, fibro-osseous lesion, juvenile ossifying fibroma, psammomatoid, trabecular

## Abstract

Juvenile ossifying fibroma (JOF) is an uncommon fibro-osseous lesion occurring in the facial bones. It is highly aggressive and has a strong tendency to recur. It has been recognized as a separate histopathological entity among the fibro-osseous group of lesions. Surgical resection is the preferred line of treatment. Here we report two cases of JOF who reported to the oral medicine and radiology department; the two cases had different clinical features, history, radiological appearance, and aggressiveness. Under the recent classification system, both cases were recognized as histopathological variants of JOF: one psammomatoid and the other trabecular.

Juvenile ossifying fibroma (JOF) is a fibro-osseous lesion that occurs in the facial bones.[1] It is also called aggressive ossifying fibroma due to its aggressiveness and the high tendency to recur, unlike other fibro-osseous lesions, such as cemento-ossifying fibroma, which it may resemble radiographically. Due to its distinct histological features, it has been recognized as a separate histopathological entity among the fibro-osseous group of lesions.[1–3] A recent study by El-Mofty[4] identified two histopathological variants, trabecular JOF (TrJOF) and psammomatoid JOF (PsJOF).

## Case Reports

### Case 1

**Figure 1:**
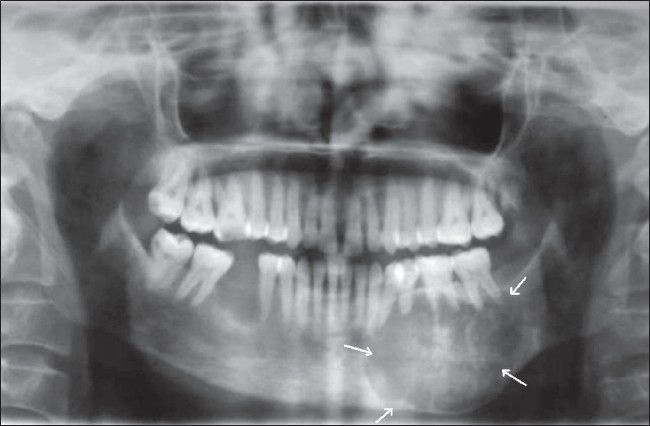
Orthopantomogram (OPG) shows a well-defined multilocular radiolucency (arrows) extending from teeth 34 to 37

**Figure 2:**
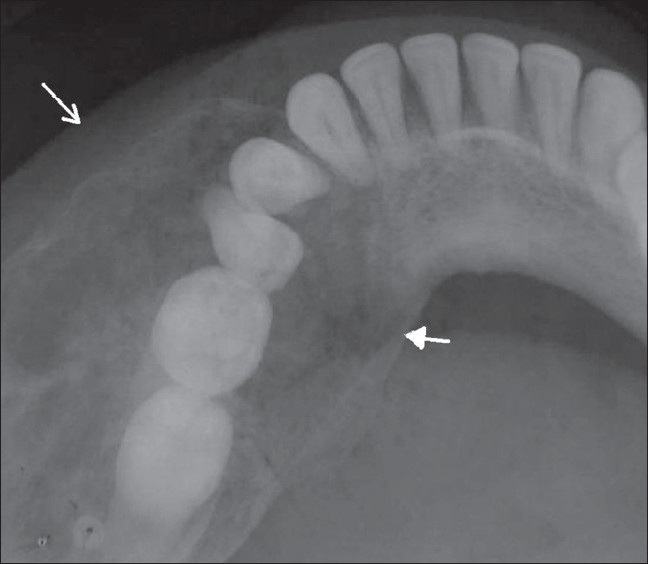
Occlusal view shows expansion of the buccal (arrow) and lingual (arrowhead) cortical plates

**Figure 3:**
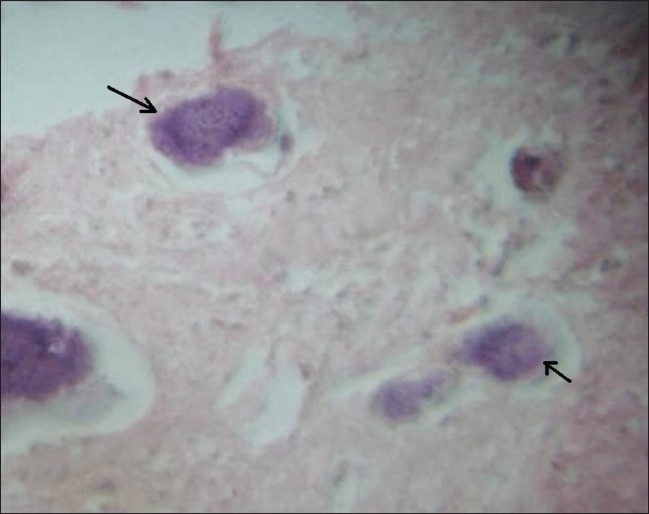
Hematoxylin and eosin–stained section shows psammoma osteoids (arrows)

A 27-year-old woman came to the oral medicine department with a swelling on the left side of the face, which had started as a small lump 2 years back but had shown a rapid increase in size over the last 6 months. There was a bony-hard swelling on the body of the left half of the mandible. On intraoral examination, there was buccal and lingual cortical expansion from teeth 32-37, with absence of the third molar. There was no history of extraction. There was no history of pain, toothache, discharge of pus, or paresthesia. Periapical and the occlusal radiographs showed a well-defined, 5 × 4 cm, multilocular radiolucency extending from teeth 34 to 37 [Figure 1]. There was no root resorption, but the premolar roots were displaced. There were two faint septae running through the inferior part of the lesion. There was gross expansion of both the buccal and lingual cortical plates [Figure 2]. The lesion showed subtle radiopaque flecks. The whole lesion showed a centrifugal growth pattern with downward bowing of the inferior cortex of the mandible. Incisional biopsy showed the lesion to be a psammomatoid type of JOF [Figure 3]. The affected portion of the mandible was resected and reconstructed. The patient is under follow-up.

### Case 2

**Figure 4:**
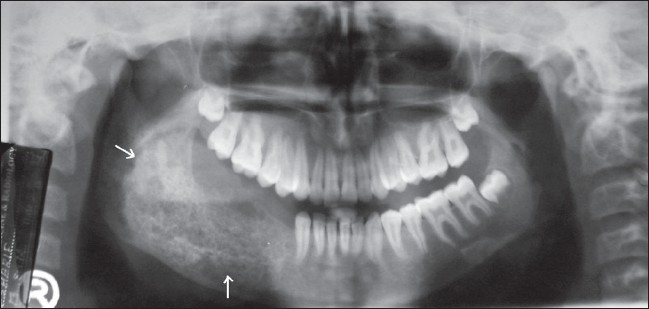
OPG shows a mixed radiopaque–radiolucent lesion (arrows) involving the body and ramus of the mandible

**Figure 5:**
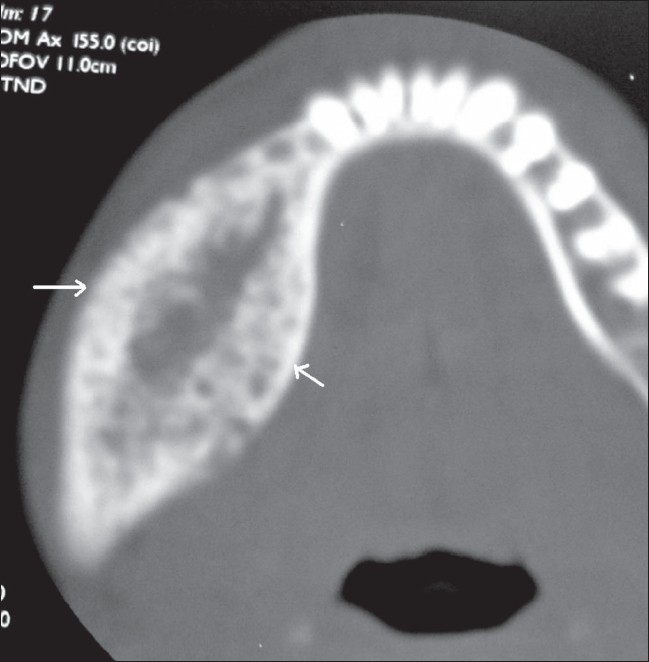
Axial CT scan (bone window) shows an expansile, osteolytic lesion (arrows), with gross expansion of both cortical plates

A 13-year-old boy presented to the oral medicine department with a painless swelling of two months' duration over the right posterior mandibular region. He had been treated for a JOF 3 years ago, with curettage and extraction of all the teeth in that region. He had been on regular follow-up and at his last visit, 3 months prior to presentation, no abnormality had been found. On presentation, he had a bony-hard swelling over the right side of the mandibular body. Intraoral examination showed buccal and lingual cortical plate expansion, extending from the distal aspect of the canine to the anterior border of the ramus. A panoramic radiograph showed a 7 × 3 cm, ill-defined, mixed radiopaque-radiolucent lesion that extended from tooth 43 to the ramus region superiorly, almost up to the sigmoid notch [Figure 4]. CT scan showed an expansile, osteolytic lesion with gross expansion of both cortical plates [Figure 5].

**Figure 6:**
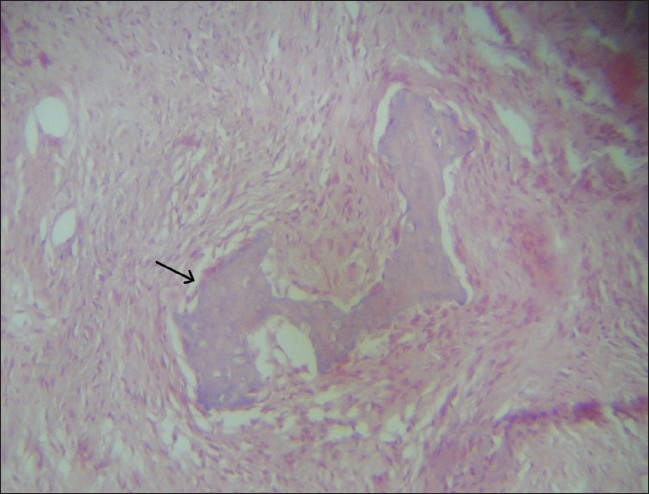
Hematoxylin and eosin–stained section shows trabecular osteoid (arrow)

An incision biopsy showed a TrJOF [Figure 6]. The affected portion of the mandible was resected and this was followed by reconstruction. The patient is under strict follow-up and no recurrence has been reported so far.

## Discussion

The most characteristic feature of JOF, as the name suggests, is its higher incidence in children and young adults.[5,6] However, it can also occur in the older age-groups.[4] Johnson *et al*.[5] have reported JOFs occurring at any age between 3 months and 72 years. Among the many classification systems for this lesion, the classification by Slootweg *et al*.[7] is noteworthy. They have classified JOF into two distinct groups, the JOF-WHO type and JOF-PO (psammoma-like ossicles) type, based primarily on the difference in the age of occurrence: the mean age of occurrence of JOF-WHO is 11.8 years and that of JOF-PO is 22.6 years.[7] The most recent classification is by El-Mofty[4] who identified two categories, trabecular JOF (TrJOF) and psammomatoid JOF (PsJOF), based on histologic criteria. However, the two categories also have a distinct predilection for specific age-groups: the average age of occurrence of TrJOF is 8½–12 years, whereas that of PsJOF is 16–33 years.[4]

Although JOF can occur anywhere in the skeleton, its highest incidence is in the facial bones, most commonly the maxilla.[3–5,8] One clinical feature that helps differentiate TrJOF from PsJOF is the site of involvement, with PsJOF occurring mainly in the paranasal sinuses and TrJOF occurring mainly in the maxilla.[4] Mandibular and extracranial involvement are rare.[5]

Gender predilection has been a matter of controversy, with some authors claiming no predilection for either sex, whereas Johnson *et al*. found a higher incidence in females[3,5] and El-Mofty reported a male predilection.[4]

JOF usually manifests as an asymptomatic bony-hard swelling, the duration and extent of which may vary depending on the site and aggressiveness of the lesion; however, it does not demonstrate the chronic, long-standing evolution of some of the other fibro-osseous lesions. It can expand the involved bones, causing facial asymmetry. Depending on the site, symptoms such as pain, paresthesia, malocclusion, sinusitis, proptosis, etc., can also occur due to the swelling.[8,9]

Radiographically the internal structure can be radiolucent, mixed, or radiopaque, depending on the degree of calcification.[3,4] Root displacement is common and resorption, though rare, can occur.[4,8,9] The lesion can cause expansion as well as perforation.[4] The radiographic features of JOF can resemble that of other lesions, such as fibrous dysplasia and cemento-ossifying fibroma.[8,10] JOF is not capsulated but is separated from surrounding bone by a radiopaque border,[3,8] and this finding can help in differentiating it from fibrous dysplasia.[3,10] A ‘ground-glass’ appearance on radiographs has been reported.[4] It usually has a concentric or centrifugal growth pattern, which can lead to an erroneous clinical diagnosis of cemento-ossifying fibroma.[8,10] JOF has also been reported to be associated with other bony lesions such as aneurysmal bone cyst.[4] Aggressive lesions with marked destruction of adjacent structures may radiographically mimic osteogenic sarcoma.[8,9]

The microscopic features of the lesion are distinctive and include a cell-rich fibrous stroma containing bands of cellular osteoid without osteoblastic lining, osteoid strands, and trabeculae of woven bone.[4,7,10] PsJOF is slightly more cellular than TrJOF. Due to the resemblance of the psammoma-like ossicles seen in PsJOF to the cementicles in cemento-ossifying fibroma, it has been argued that PsJOF is a type of cemento-ossifying fibroma.[2,4] However, the marked cellularity of JOF is in sharp contrast to the usually stroma-rich appearance of the latter group of lesions.

The aggressive nature of this entity, along with the reported high rates of recurrence (30–58%),[4,8] suggests that JOF should be treated like a locally aggressive neoplasm, very much like an ameloblastoma. Surgical resection, rather than conservative curettage, is therefore the preferred line of treatment.[6,8]

The incidence of JOF is still unknown because of the relatively few cases reported till date. We have reported two cases of JOF, each different in its clinical, radiological, and histopathologic aspects. The first case mimicked cemento-ossifying fibroma clinically as well as radiologically and had a slow growth over more than 2 years. Histologically it was seen to be psammomatoid JOF, which is uncommon in the jaws, especially in the mandible.

The second case deserves attention because of the fact that the lesion recurred after initial conservative management, thus demonstrating the tendency of this disease to recur. Moreover, the aggressiveness of the recurrent lesion can be gauged from the rapid growth it showed over a period of less than 3 months. Histopathologically, this case was a trabecular JOF. This case supports the earlier literature[5,8] that argues in favor of segmental resection of the involved area to avoid recurrence. The age of occurrence of both the cases is consistent with the description given by El-Mofty.[4]
